# Prevalence and determinants of effective breastfeeding technique among early postpartum mothers in Fuzhou, China: A cross-sectional study

**DOI:** 10.1371/journal.pone.0319408

**Published:** 2025-02-25

**Authors:** Neema E. Mawi, Hu Rong-Fang, Heavenlight A. Paulo, Na Chen, Gui-Hua Liu, Mohammed Abba-Aji

**Affiliations:** 1 Department of Midwifery, School of Nursing, Fujian Medical University, Fuzhou, Fujian, China; 2 Department of Epidemiology and Biostatistics, School of Public Health and Social Sciences, Muhimbili University of Health and Allied Sciences, Dar es Salaam, Tanzania; 3 Department of Epidemiology, School of Public Health, Boston University, United States; Ateneo de Manila University Ateneo School of Medicine and Public Health, PHILIPPINES

## Abstract

**Background:**

Breastfeeding technique (BFT) is determined by the mother’s and infant’s positioning, the infant’s attachment to the breast, and the infant’s suckling behavior. Understanding breastfeeding mothers’ skills is crucial for clinical practice and for designing interventions to improve breastfeeding practices. This study aimed to determine the prevalence and determinants of effective BFT among early post-partum mothers.

**Methods:**

We conducted a cross-sectional study among 415 early post-partum mothers in Fuzhou, China. Participants were recruited using a systematic random sampling technique. Data were collected using a self-administered questionnaire and a standardized observational checklist (WHO B-R-E-A-S-T Feed observation form). Descriptive statistics were used to assess the prevalence of effective BFT, and logistic regression was applied to identify its determinants.

**Results:**

The overall prevalence of effective BFT was 70.4%. After adjusting for potential confounders, the prevalence ratio (PR) of effective BFT was greater among participants with college (PR = 1.20) and postgraduate education (PR = 1.41) compared to those with a technical education or lower. Participants who attended antenatal care (ANC) (PR = 1.04) and those with BFT knowledge (PR = 1.37) were more likely to practice effective BFT compared to participants who did not attend ANC and without such knowledge respectively. Similarly, those who received BFT counseling during pregnancy or immediately after delivery were 25% and 30%, respectively, more likely to practice effective EBT than those who did not. Conversely, experiencing breast problems was associated with a 42% lower likelihood of practicing effective BFT than those without breast problems.

**Conclusion:**

Effective BFT is prevalent among early postpartum mothers in Fuzhou, China, with education level, antenatal care attendance, knowledge, and counseling playing significant roles in its practice. Efforts to improve breastfeeding outcomes should focus on enhancing educational interventions and providing targeted counseling during pregnancy and the immediate postpartum period, while addressing breast health issues to mitigate their impact on breastfeeding effectiveness.

## Background

Breastfeeding provides numerous benefits for both mothers and children, including protection against various acute and chronic illnesses [1]. In 2019, approximately 2.7 million children died from malnutrition; however—more than 820,000 of these deaths could have been prevented if children under two years old had been optimally breastfed [2]. The World Health Organization (WHO) recommends exclusive breastfeeding (EBF) for the first six months of life, followed by the introduction of complementary foods alongside continued breastfeeding until 24 months of age [3].

Breastfeeding technique (BFT) is determined by the mother’s and infant’s positioning, the infant’s attachment to the breast, and the infant’s suckling [4]. Ineffective BFT can result in inadequate milk drainage, leading to conditions such as breast engorgement and mastitis [5,6]. These conditions can increase the likelihood of early weaning or breastfeeding cessation due to the pain and discomfort experienced by mothers [7,8]. Research indicates that mothers who experience breast-related complications or other difficulties during breastfeeding, especially within the first month postpartum, are more likely to wean their children earlier compared to those who do not encounter such challenges [9–11].

The prevalence of effective BFT ranges from 42.2%–64% based on studies conducted in Sri-Lanka, Brazil, Ethiopia, and Nigeria [4,12–14]. The practice of effective BFT is influenced by maternal sociodemographic factors, as well as access and utilization of healthcare services [4,14–16]. Moreover, breastfeeding experience, breastfeeding counseling, breast problems, and BFT knowledge also contribute to effective BFT [12,17–19]. Infant factors such as age, birth weight, and gestational age (GA) have also been reported to influence BFT [20,21].

Despite the available evidence on the impact of BFT on breastfeeding practices, the practice of effective BFT in China, particularly Fuzhou, remains underexplored. Moreover, gaps persist in our understanding of the impact of mode of delivery on the practice of effective BFT. Some studies suggest that spontaneous vaginal delivery (SVD) increases the likelihood of ineffective BFT [15,21] while other studies report the opposite [16,22]. Additionally, most studies have been conducted after postpartum mothers were discharged from the hospital [4,19,23]. Assessing postpartum mother’s breastfeeding practice before hospital discharge is crucial to reducing breastfeeding challenges, identifying and addressing existing difficulties, and equipping them with the necessary breastfeeding knowledge and skills. This is crucial for guiding interventions aimed at improving BFT and, subsequently enhancing breastfeeding practice.

To address the existing evidence gap, we sought to assess the prevalence of effective BFT and its associated factors among early post-partum mothers in hospitals following delivery in Fuzhou, China. To achieve this aim, we conducted a cross sectional study and we hypothesized that multiple factors including mother’s and infant’s socio-demographic characteristics, obstetric history, maternal knowledge about BFT and breastfeeding self-efficacy are associated with practice of effective BFT.

## Materials and methods

### Study design and setting

We conducted a hospital based cross-sectional study at a public maternal and child hospital in Fuzhou, Fujian province, China. The hospital provides specialized maternal care and immunization services for both in and outpatients. Approximately 21,700 deliveries occur annually at this hospital. Data were collected from September to December 2021.

### Study population and sampling

The participants were post-partum mothers in the postnatal wards. The inclusion criteria were mothers: (1) who gave birth to a live singleton baby by SVD or Caesarean Section (CS), (2) without serious health conditions, mental illness, or communication difficulties, and (3) who had initiated breastfeeding at the time of data collection (within 12 hours for post-SVD or 24 hours for post-CS). The exclusion criteria were: mothers unable to breastfeed because of medical conditions or contraindications; (2) sick infants, or those who refused to feed, and; (3) infants with palate or tongue deformities.

The sample size was determined by using a formula for a single population proportion for a cross-sectional study [24] based on the following statistical assumptions: the prevalence of effective BFT 43.4% [4], 95% level of confidence, and 5% margin of error. A total sample of 415 was calculated after accounting for a 10% non-response rate. A systematic sampling technique was used to recruit study participants. The sampling interval (k) was obtained by dividing the total number of deliveries one month before data collection by the required sample size (1441/415 = 3.4). Therefore, participants were selected after every third postpartum mother until the required sample size was reached.

### Variables and instruments

The data were collected using self-administered questionnaires and a standardized observational checklist-WHO B-R-E-A-S-T Feed observation form [1]. The questionnaire were adapted from previous literature [4] and modified to meet the objectives of this study. The primary researcher (NM) in collaboration with research assistant (Na, C) translated the English version of the questionnaire into Chinese language which was later refined by research supervisor to ensure consistency and culturally appropriateness. The questionnaire gathered information on maternal and infant demographics, obstetric history, BFT knowledge, and breastfeeding self-efficacy.

### Dependent variable

#### BFT assessment.

The primary outcome of this study was practice of BFT. BFT is a composite of three variables: mother and infant positioning, infant’s attachment to the breast, and effective suckling. A standardized WHO B-R-E-A-S-T Feed observation form was used to observe and record BFT [1]. There are a total of 11 items on the form (position and attachment each included four items, and suckling that included three items), and the responses were recorded as “Yes” for correct technique and “No” for incorrect technique. Positioning was considered to be “Poor” when the score was “0–1,” “Average” when the score was “2,” and “Good” when the score was “3–4”. Likewise, attachment was considered to be “Poor” when the score was “0–1,” “Average” when the score was “2”, and “Good” when the score was “3–4”. Suckling was considered to be “Poor” when the score was “1,” and “Good when the score was “2–3”. BFT was categorized as either “effective BFT” if the composite index of the three mentioned variables was ≥ 7 or “ineffective BFT” if the composite index was <7 [4].

#### Independent variables.

The independent variables for our study included BFT knowledge, breastfeeding self-efficacy as well as maternal and infant demographic and obstetric history. These variables were measures as follows:

#### BFT knowledge assessment.

Primary researcher (NM) developed assessment tool to measure this variable, that was built upon previous study [1,17]. The tool comprised 18 items, each designed to evaluate all three key aspects of BFT. Participants’ responses were categorized as either “correct,” “incorrect,” or “I don’t know.” Each correct response was scored as “1,” while an incorrect and “I don’t know” responses were scored as “0.” The result overall score ranged from 0 to 18, with a higher score indicating a higher level of knowledge. Notably, the assessment tool employed in the current study demonstrated a commendable test-retest reliability value (r) of 0.874.

#### Breastfeeding Self-Efficacy Assessment.

To gauge this variable, the Breastfeeding Self-Efficacy Scale-Short Form (BSES-SF) was utilized. This tool was translated and validated to Chinese mothers in a previous study [25]. The tool consisted 14 positively worded items in a 5-points Likert scale ranging from 1 (indicating “not at all confident”) to 5 (indicating “always confident”). The cumulative BSES-SF scores spanned from 14 to 70, where a higher score indicated greater maternal confidence in breastfeeding. Prior to its use in the study, the tool underwent pre-testing with 5% of the research participants, yielding an impressive Cronbach’s alpha score of 0.94.

#### Participants’ demographic and obstetrics information.

A total of 38 items was used to gather data on maternal and infant demographic and obstetrics characteristics. Infant’s demographic characteristics were age, sex, birth weight, and GA while maternal demographic characteristics comprised age, ethnicity, religion. Obstetric history included parity, ANC attendance, maternity school attendance, mode of delivery, BFT counseling during pregnancy, BFT counseling immediately following delivery, breast problems, support received during pregnancy, and pregnancy status (planned or unplanned pregnancy).

### Data collection

Participants were informed of the purpose of the study, benefits and risk of participating and right to withdraw before signing the consent form. They completed the questionnaire first, followed by BFT observation, where mothers were requested to breastfeed if an hour had passed since their last feeding. If feeding had occurred within the hour or the infant refused to suckle, an agreement for the next feeding was arranged. Observations were conducted for 3–4 minutes at the participant’s bedside with maximum privacy.

### Data analysis

Data cleaning and analysis were conducted using STATA version 15 (StataCorp. 2017. Stata Statistical Software: Release 15. College Station, TX: StataCorp LLC.). Shapiro-Wilk test was used to assess the distribution of the continuous variables, with results summarized using median and interquartile range (IQR). Frequencies and percentages summarized categorical variables. A modified Poisson regression model with a robust standard error and log link function estimated risk ratios (RR) was used to identify factors associated with effective BFT. To control for potential confounding factors, variables with a p-value < 0.20 in crude analysis were included in a multivariable model. Variables included in the model were maternal age, education status, residence, parity, ANC attendance, mode of delivery, BFT counseling during pregnancy and immediately after delivery, breast problems, and knowledge about BFT. An Adjusted prevalence ratio (APR) with 95% confidence intervals (CI) and a *p*-value threshold of <  0.05 was used to determine significant associations with effective BFT.

### Ethical approval and consent to participate

Ethical approval, reference number 2021YJ044, and permission to conduct a study were obtained from Fujian Provincial Maternity and Children’s Hospital Research Ethics Boards. All eligible participants provided written informed consent. To maintain confidentiality, participants were identified by unique code numbers, which were used during data entry, cleaning, and further processing.

## Results

### Background characteristics of participants.

Table 1 presents the background characteristics of 415 participants in the study. The median age (IQR) of participants was 30.0 (28.0–33.0) years. The majority of participants were Ethnic Han (98.6%). Based on educational attainment and working status, 73.0% had college education and 81.4% were working. Most participants were married (98.8%) and 69.2% lived in cities. More than half of the participants’ 273 (65.8%) had monthly family income below 10000¥. The average family size (IQR) was 3.0 (3.0–4.0) people.

**Table 1:** Background characteristics of participants (N = 415).

Variables	n (%)/ Median (IQR)
**Age (years)** ^†^	30.0 (28.0–33.0)
**Ethnicity**	
Ethnic Han	409 (98.6)
National Minority	6 (1.4)
**Religion**	
No religion	263 (63.4)
Buddhist	110 (26.5)
Christian	41 (9.9)
Hindu	1 (0.2)
**Education status**	
Technical or below education	80 (19.3)
College education	303 (73.0)
Postgraduate education	32 (7.7)
**Occupation**	
Housewife	77 (18.6)
Government employee	145 (34.9)
NGO’s/Private employee	158 (38.1)
Self-employee	35 (8.4)
**Marital status**	
Married	410 (98.8)
Cohabiting	1 (0.2)
Single	4 (1.0)
**Residence**	
Rural area	128 (30.8)
City	287 (69.2)
Monthly family income (CNY)*	
≤10000	273 (65.8)
>10000	142 (34.2)
**Family size** ^†^	3.4 (3.0–4.0)

^†^Continuous variables,

*Exchange rate during data collection, 1USD =6.4529 CNY.

### 3.2 Maternal obstetrics and infant’s characteristics

About half of the participants (52.3%) were primiparous and 57.6% gave birth vaginally. Most attended ANC follow-up (89.4%) and 59.0% attended maternity school. Majority of the participants (83.9%) and 86.0% received BFT counseling during the pregnancy period and immediately after delivery, respectively. Only 20.7% experienced breast problems. Regarding the characteristics of the infants, 98.8% were delivered at term and 95.7% had normal birthweights. About a third (31.8%) of the infants received a pre-lacteal meal and two-third (65.8%) started complementary feeding (Table 2). Reasons for pre-lacteal meal provision and early weaning are shown in Table 3.

**Table 2:** Obstetrics characteristics of the participants (N = 415).

Variables	n (%)/Median (IQR)	Variables	n (%)/Median (IQR)
**Parity**		**Cadre of the HCP** ^1^	
Primipara	217 (52.3)	Nurses only	60 (20.5)
Multipara	198 (47.7)	Doctors only	71 (24.3)
**Mode of delivery**		Nutritionists only	21 (7.2)
SVD	239 (57.6)	Multi-professions	140 (48.0)
CS	163 (39.3)	**Received postnatal counseling about BFT**	
Assisted delivery	13 (3.1)	Yes	357 (86.0)
**Pregnancy status**		**Cadre of the HCP** ^2^	
Planned	269 (64.8)	Nurses only	193 (54.1)
**Received support during pregnancy**		Nutritionists only	9 (2.5)
Yes	401 (96.6)	Multi-professions	111 (31.1)
**Support provider**		**Breast problems**	
Partner	365 (88.0)	Present	85 (20.7)
Parents	304 (73.3)	**Type of a problem**	
Parents in-laws	286 (68.9)	Crackle nipple	43 (50.6)
Relatives	134 (32.3)	Mastitis	2 (2.4)
Friends	107 (25.8)	Engorgement	8 (9.9)
Other providers^a^	5 (1.2)	Inverted nipple	33 (38.8)
**ANC attendance**		Other^c^	5 (5.9)
Yes	371 (89.4)	**Gestation age at delivery (weeks)**	
**Frequency of ANC attendance**		Preterm (<37 weeks)	5 (1.2)
1–3 times	49 (11.8)	Term (37–42 weeks)	410 (98.8)
≥4	322 (77.6)	**Sex of the infant**	
**Maternity school attendance**		Male	212 (51.1)
Yes	244 (58.8)	Female	203 (48.9)
**Frequency of maternity school attendance** ^†^	1.0(0.0–1.0)	**Infant weight (kg)**	
**Breastfeeding experience**		Low birth weight (*<*2.5 kg)	8 (1.9)
Yes	337 (81.2)	Normal birth weight (2.5 kg–4 kg)	397 (95.7)
**BFT counseling during pregnancy**		Macrosomia (>4 kg)	10 (2.4)
Yes	348 (83.9)	**Introduce pre-lacteal meal to infant**	
**Provider of BFT counseling during pregnancy**		Yes	132 (31.8)
Healthcare professionals only	163 (46.8)	**Initiate complementary feed**	
Healthcare professionals + others ^b^	185 (53.2)	Yes	273 (65.8)

^†^Continuous variable,

^a^Colleagues,

^b^Parents, husband, relatives, friends, self-learning, books, internet, WeChat, and video clips,

^c^Breast nodules, breast lumps, breast hyperplasia,

^1^Provider of BFT counseling during pregnancy,

^2^Provider of BFT counseling after delivery.

**Table 3:** Reasons for providing pre-lacteal meals and initiating complementary feed.

Variables	Reasons	n (%)
**Introduce pre-lacteal meal to infant (n = 132)**	Delayed milk secretion	80 (60.6)
	Culturally practiced	3 (2.3)
	Advised by the healthcare provider	21 (15.9)
	Suggestions from family members and friends	11 (8.3)
	Worried baby might be hungry or thirst	44 (33.3)
	Other reasons (not specified)	3 (2.3)
**Initiate complementary feed (n = 273)**	Breast milk is not sufficient	238 (87.2)
	Culturally practiced	4 (1.5)
	Advised by the healthcare provider	25 (9.2)
	Worried about baby’s nutrition	41 (15.0)
	Other reasons (not specified)	7 (2.6)

#### Prevalence of effective breastfeeding technique.

**Fig 1:**
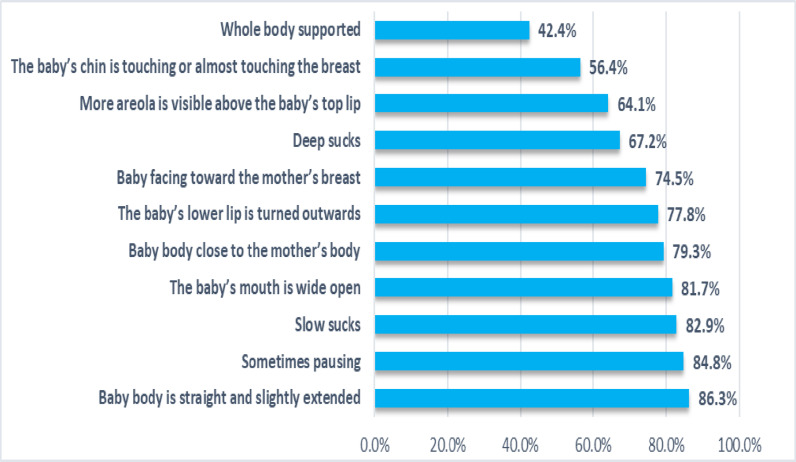
Effective breastfeeding practice for each item (N = 415).

Within the study population, the overall prevalence of effective BFT was 70.4%. About two thirds of the participants exhibited good positioning 66.5% (276) and good attachment 60.2% (250). Further, 80.0% (332) of the infants demonstrated effective suckling. Of the 11 items assessed, the majority of mothers (86.3%) held their baby’s body straight and slightly extended, while only 42.4% supported the entire body of their infants (Fig 1).

#### Factors associated with effective breastfeeding technique.

Our multivariable analysis reveals that when compared to participants with a technical or below education level, mothers with college (APR: 1.20; 95% CI: 1.00, 1.46) and postgraduate education degrees (APR: 1.41; 95% CI: 1.12, 1.76) had greater levels of practicing effective BFT. Furthermore, participants who actively engaged in ANC follow-up (APR: 1.37; 95% CI: 1.04, 1.79) and those who had knowledge about breastfeeding (APR: 1.04; 95% CI: 1.01, 1.07) had greater level of performing effective BFT. Similarly, participants who received BFT counseling during pregnancy (APR: 1.25; 95% CI: 1.02, 1.53) as well as immediately post-delivery (APR: 1.38; 95% CI: 1.07, 1.77) were more likely practice effective BFT compared to those who hadn’t received such counselling. Additionally, mothers experiencing breast problems had a 42% reduced likelihood of practicing effective BFT (APR: 0.58; 95% CI: 0.45, 0.73) when compared to those without breast problems (Table 4).

**Table 4:** Multivariable analysis of factors associated with effective BFT among participants (N = 415).

Variables	Effective BFT	CPR (95% CI)	*p-value*	APR (95% CI)	*p-value*
	Yes (N, %)				
**Mothers age** ^†^		1.02 (1.00, 1.03)	***0.030***	1.01 (1.00, 1.02)	*0.379*
**Education status**					
Technical or below education	46 (57.5)	1		1	
College education	217 (71.6)	1.25 (1.02, 1.52)	***0.033***	1.20 (1.00, 1.46)	***0.044***
Postgraduate education	29 (90.6)	1.58 (1.27, 1.96)	***<0.001***	1.41 (1.12, 1.76)	***0.003***
**Residence**					
Rural area	78 (60.9)	1		1	
City	214 (74.6)	1.22 (1.05, 1.43)	***0.010***	1.06 (0.92, 1.23)	*0.418*
**Family size** ^†^		0.97 (0.85, 1.10)	*0.619*		
**Parity**					
Primipara	138 (63.6)	1		1	
Multipara	154 (77.8)	1.22 (1.08, 1.39)	***0.002***	1.11 (0.98, 1.26)	*0.103*
**ANC attendance**					
Yes	271 (73.0)	1.53 (1.12, 2.10)	***0.008***	1.37 (1.04, 1.79)	***0.023***
No	21 (47.7)	1		1	
**Frequency of maternity school attendance** ^†^		1.01 (0.95, 1.08)	*0.792*		
**Mode of delivery**					
SVD	185 (73.4)	1		1	
CS	107 (65.6)	0.89 (0.78, 1.02)	*0.091*	0.92 (0.81, 1.04)	0.162
**Received BFT counseling during pregnancy**					
Yes	256 (73.6)	1.37 (1.09, 1.73)	***0.008***	1.25 (1.02, 1.53)	***0.035***
No	36 (53.7)	1		1	
**Received postnatal counseling about BFT**					
Yes	264 (73.9)	1.53 (1.16, 2.01)	***0.002***	1.38 (1.07, 1.77)	***0.012***
No	28 (48.3)	1		1	
**Breast problems**					
Yes	37 (43.5)	0.56 (0.44, 0.72)	***<0.001***	0.58 (0.45, 0.73)	***<0.001***
No	255 (77.3)	1		1	
**Knowledge about BFT** ^†^		1.05 (1.02, 1.09)	***0.001***	1.04 (1.01, 1.07)	**0.005**
**Breastfeeding efficacy** ^†^		1.00 (1.00, 1.01)	*0.304*		

^†^Continuous variable, CPR-Crude Prevalence Ratio, APR-Adjusted Prevalence Ratio, CI-Confidence interval.

## Discussion

This study aimed to assess the prevalence and determinants of effective BFT among early post-partum mothers in a hospital setting in Fuzhou, China. We found that 70.4% of women delivering in the study site practiced effective BFT post-delivery. Several individual-level factors, including education level, antenatal care (ANC) attendance, breastfeeding problems, and BFT knowledge, as well as healthcare access factors such as BFT counseling during and after birth, were identified as key determinants.

The prevalence of effective BFT observed in our study was higher compared to findings from previous studies conducted in Denmark, Brazil, and Ethiopia [13,14,19]. The quality of services offered at the current study site, combined with patient characteristics, may explain these findings [26]. The hospital mandates that pregnant women participate in essential courses, including a breastfeeding course. This course covers topics such as proper feeding and breastfeeding techniques, delivered either through in-person classes or online videos, based on individual preferences. This suggests that maternity patients at this hospital have greater access to high-quality prenatal education, including breastfeeding skills training. Previous studies have shown that women who received training on BFT demonstrated better positioning and attachment during breastfeeding compared to those who did not receive such training [13,27]. Further, the higher cost healthcare services at this advanced-level hospital may naturally attract clients with higher incomes. Over three-quarters of the study participants reported a monthly income exceeding the regional minimum wage of $286 [28]. Although no direct association was found between income and BFT, higher family income may have indirectly influenced the practice, as women with greater financial resources were more likely to afford the hospital’s services.

We found that participants with higher levels of education practiced effective BFT more commonly than those with lower levels of education. These findings are consistent with those of studies conducted in Saudi Arabia, India, and Ethiopia [4,16,29], and may be explained by the correlation between a high level of education and breastfeeding knowledge and a positive intention to breastfeed [30,31]. Such positive intentions to breastfeed may drive mothers to acquire additional breastfeeding knowledge and skills [4]. In addition, participants with lesser levels of education may need additional time to grasp information and learn given skills.

Our study also found that participants who attended ANC exhibited greater effective BFT compared to those who had not. This finding, similar to that of a study from Nigeria, could be as a result of breastfeeding and infant feeding counseling offered during ANC visits [12]. Such counselling sessions aim to improve breastfeeding self-efficacy, establish healthy feeding habits, and resolve breastfeeding challenges [32,33]. In previous studies, it was found that using ANC enhanced the practice of EBF [34]. Therefore, an effort to promote ANC use and thereby enhance breastfeeding is still necessary.

We found that breast problems such as nipple cracks, breast engorgement and mastitis were negatively associated with effective BFT practices. Mothers with breast problems were 42% less likely to practice effective BFT compared to their counterparts without such problems. This finding consistent with those from studies conducted in Pakistan and Ethiopia [14,35] can be attributed to the pain and discomfort experienced by breastfeeding mothers with breast problems. Breast problems are a significant barrier to breastfeeding. However, an interventional study conducted in China reported promising evidence on prevention of these problems through provision of breastfeeding education guidance, using aids like dolls and breasts for postpartum mothers [36]. Moreover, mothers with breast problems require extra assistance to overcome existing problems, master proper breastfeeding skills and encouragement to continue with lactation. Therefore, this study stresses the importance of a regular assessment of postpartum mothers particularly in the first few weeks postpartum to identify breastfeeding problems and manage them accordingly.

We found that greater BFT knowledge was associated with an increase in the likelihood of effective BFT. This finding is consistent with studies conducted in Nepal and India [18,37], which reported a positive association between BFT knowledge and the practice. A systematic study conducted in Turkey [38] identified a lack of maternal knowledge as a significant barrier to effective breastfeeding. Inadequate knowledge of BFT can lead to the adoption of ineffective techniques, resulting in musculoskeletal issues such as neck and back pain, as well as breast-related problems like nipple cracks and breast engorgement [17]. Previous studies conducted in Turkey and India recommended development and implementation of breastfeeding education and counseling interventions for pregnant and postpartum women to increase knowledge of BFT and breastfeeding skills [37,38].

Our study also found that BFT counseling offered during pregnancy and immediately following delivery increased the likelihood of mothers to practice of effective BFT. This finding aligns with studies conducted in Ethiopia and Nigeria [12,14], which underscore the importance of the prenatal and postnatal periods as critical windows for equipping mothers with positive breastfeeding attitudes and skills [39]. Further, studies show that breastfeeding counseling provided during prenatal period could reduce some breastfeeding challenges that breastfeeding mothers face during the postpartum period [33,38].

This study has several limitations that should be considered. Due to its cross-sectional design, it was not possible to establish a temporal relationship between some independent and dependent variables. Additionally, the use of observational checklists may have introduced observer bias; however, efforts to minimize this were made by training data collectors for four months in the postnatal ward before data collection. Furthermore, as the study was conducted in a single hospital, the findings may not be generalizable to other settings. Future studies should consider larger sample sizes and diverse healthcare settings to enhance the generalizability of findings.

## Conclusion

The majority of mothers in this study practiced effective BFT and key factors such as sociodemographic characteristics, health status, and healthcare access were significantly associated with effective BFT. BFT knowledge and counseling during pregnancy and immediately postpartum had a substantial impact on effective BFT. Encouraging ANC attendance and providing breastfeeding counselling, particularly for primiparous mothers, can improve BFT practices. Healthcare providers should conduct regular postpartum assessments to identify and address breastfeeding challenges in a timely manner.

## Supporting information

S1 File English questionnaire. (PDF)

S2 File Swahili questionnaire. (PDF)

S3 Dataset Analysed dataset. (DTA)

## Abbreviations

ANC: Antenatal Care
APR: Adjusted Prevalence Ratio
BFH: Baby Friendly Hospital
BFT: Breastfeeding Technique
BSES-SF: Breastfeeding Self-Efficacy-Scale Short Form
CI: Confidence Interval
CPR: Crude Prevalence Ratio
CS: Cesarean section
GA: Gestation Age
EBF: Exclusive Breast Feeding
IMNCI: Integrated Management of Neonate and Childhood Illness (IMNCI)
KAP: Knowledge Attitude and Practice
LATCH: Latch into the breast; Audible swallowing; Type of nipples; Comfort level; and Help needed by mother to hold a baby to the breast
NGO: Non-Government Organization
PNC: Post-natal Care
SD: Standard Deviation
SVD: Spontaneous virginal delivery
UNICEF: United Nations Children’s Fund
WHO: World Health Organization
WHO B-R-E-A-S-T: World Health Organization Breastfeeding.
