# Supplementary material for: Prevalence and determinants of effective breastfeeding technique among early postpartum mothers in Fuzhou, China: A cross-sectional study
Source: PLoS One. 2025 Feb 25;20(2):e0319408. doi: 10.1371/journal.pone.0319408 (PMC11856331; doi:10.1371/journal.pone.0319408)
Supplement: S1 File — (PDF) [file pone.0319408.s001.pdf]

ENGLISH QUESTIONNAIRE

Serial number:____________

Hello, I am a graduate student from Fujian Medical University. We are doing research on topics related to breastfeeding. Thank you very much for taking the time to fill in this questionnaire. Please fill in according to your own situation and truthfully fill in.

Congratulations on becoming a great mother, I wish you good health, happy growth of the child!

Question Guide: There are several answer requirements for each question in this questionnaire. Please set the requirements according to the questions and answers, and select or fill in the options that are consistent with you.

Part-I Socio Demographic Characteristics

Instructions: Please fill in the underlined text on the space provided. For other items with options, please tick "√" in the appropriate options.

1. How old (in years) are you?___________
2. What is your nationality?
   A. Ethnic Han B. National minority C. Other, please specify
3. Do you have any religion?
   A. No religion B. Buddhist C. Christian D. Muslim E. Hindu
   F. Other, please specify ________
4. What is your relationship status?
   A. Married B. Cohabiting C. Single D. Divorced E. Widowed/widower
5. What is your occupation?
   A. House wife B. Government employee C. Non-Government Organization /Private employee D. Self-employee E. Student
6. What is your level of education?
   A. Did not go to school B. Primary education C. Junior high school
   D. Senior high school E. Technical secondary school
   F. Junior college G. Regular college course H. Postgraduate or above

7. In which part of the country, your family live?

   A. Rural areas B. Villages and towns C. City

8. Are you the only child in your family?

   A. Yes B. No

9. What is your family's monthly income?

   A. ≤2000¥ B. 2001-5000¥ C. 5001-10000¥ D. 10001-30000¥

   E. 30001-50000¥ F. ≥50001¥

10. How is your usual living environment?

    A. Quiet B. Moderate C. Noisy

11. How many hours do you sleep in each day?

    A. 6h B. 6-8h C. ＞8h

12. How is your current appetite?

    A. Worse B. Moderate C. Better

13. How many people are there in your family?

    A. 2 people B. 3 people C. 4 people D. 5 people E. ≥6 people

Part II: Obstetrics and infant characteristics

14. Including this time, how many times have you had pregnancy? _______How many times have you given birth? __________How many times have you had pregnancy not leading to birth (e.g., miscarriage, termination for medical, abortion reasons)?__________How many living children do you have?___________

15. In this pregnancy, did you participate in antenatal care in a medical institution (hospital)? If "no" skip question no. 3

    A. Yes B. No

16. How many times have you participated in antenatal care?

    A. One time B. Two times C. Three times D. Four times or more

17. In this pregnancy, have you attended the maternity school throughout? If "no" skip question no. 5

    A. Yes B. No

18. How many times did you attend the course on breastfeeding knowledge?

    A. One time B. Two times C. Three times D. Four times or more

19. Was the pregnancy planned?

A. Yes B. No

20. Did you get support during pregnancy? Including financial, family, friends, etc）If “no” skip question no. 8

A. Yes B. No

21. From who did you get support （**Check all the appropriate options√**）

A. Partner B. Father and mother C. Father and mother in law

C. Relatives i.e. sister, aunt, brother etc. D. Friends E. Others specify________

22. Have you seen other mothers’ breastfeeding their babies?

A. Yes B. No

23. Have you been taught about breastfeeding techniques during pregnancy?

A. Yes B. No

24. Whom taught you about breastfeeding techniques（**Check all the appropriate options√**）If your answer does not include option “1” skip question no. 12

A. Healthcare provider B. Parents C. Husbands D. Relatives E. Friends

F. Others specify________

25. In which industry does the healthcare provider come from? （**Check all the appropriate options√**

A. From a nurse B. From a doctor C. From a nutritionist

D. Others specify________

26. What was your delivery method for this pregnancy?

A. Normal delivery B. Cesarean section C. Assisted delivery

27. After delivery, was you taught about breastfeeding techniques immediately? If “no” skip question no. 15

A. Yes B. No

28. From whom did you get the relevant education?

A. From a nurse B. From a doctor C. From a nutritionist

D. Others specify________

29. Did you feed other foods before breastfeeding your baby?（Such as warm water, glucose water and so on）If “no” skip question no. 17

A. Yes B. No

30. Why did you feed other foods before breastfeeding? （**Check all the appropriate options**√）

A. Delayed milk secretion B. Culturally practiced C. Advised by healthcare provider D. Suggestions from family or friends E. Worried about baby hunger/thirst F. Others________

31. Have you given your child supplementary/ complementary feeding? If“ no” skip question no. 19

A. Yes B. No

32. Why do you supplement or add complementary food to your baby? （**Check all the appropriate options**√）

A. Breast milk is not sufficient B. Culturally practiced C. Advised by healthcare provider D. Worried about baby nutrition F. Others________

33. Do you have any breast problems at present? If “no” skip question no. 20

A. Yes B. No

34. If yes, please indicate the specific breast problem is

A. Crackle nipple B. Mastitis C. Engorgement D. Inverted nipple E. Other________

35. What was the gestational week when the baby was born? ________ Weeks

36. What is the baby's gender?

A. Male B. Female

37. What is the baby's weight? ___________kg

38. Age of the infant___________ hours

Part III: Knowledge about breastfeeding techniques

Instructions for filling in this form: this form describes your knowledge of breastfeeding techniques. Please tick "√" in the box where you think it is appropriate.

	Item	Correct	Incorrect	Not sure
1.	Good BFT practice benefits both mother and baby.			
2.	It is recommended that mothers lean forward or push their breasts forward toward their infants when feeding them.			
3.	Pulling the baby close to the breast by moving the arms is advisable during breastfeeding.			
4.	While breastfeeding the important infant's body parts need to be supported are head, neck and buttocks.			
5.	Several positions such as sitting, lying down and standing can be assumed by mother while breastfeeding.			
6.	More of the areola visible below the baby's bottom lip than above the top lip or the amounts above and below are equal is a sign of proper attachment.			
7.	Improper attachment includes the baby's chin being away from the breast and the baby's mouth is not wide open.			
8.	Baby's upper and lower lip everted is a sign of proper attachment.			
9.	Inverted nipple can cause poor infant attachment.			
10.	Lactating mother experiencing nipple pain or nipple cracks indicate poor baby attachment.			
11.	A baby who is poorly attached is likely to suckle ineffectively.			
12.	For effective sucking a baby have to suck slowly but deep and sometimes pauses.			
13.	A baby suckles quickly all the time without swallowing is a sign of effective suckling.			
14.	Ineffective sucking is among the cause of insufficiently			

	milk.			
15.	Breast problems such as engorgement and mastitis mostly are caused by ineffective suckling.			
16.	Engorged breast usually result to baby failure to attach properly.			
17.	Breast engorgement can be resolved by frequently feeding, warm compresses or massage.			
18.	It is not advised to breastfed a baby an engorged breast.			

Part IV: Self-efficacy about breastfeeding

Instructions for filling in this form: this form describes your knowledge of breastfeeding techniques. Please tick "√" in the box where you think it is appropriate.

**S/N**	Item	Not at all confident	Not confident	Neutral	Confident	Always confident
1.	I can always determine that my baby is getting enough milk					
2.	can always successfully cope with breastfeeding like I have with other challenging tasks					
3.	I can always breastfeeding my baby without giving other feeds					
4.	I can always ensure that my baby is properly latched on for the whole feeding					
5.	I can always manage the breastfeeding situation to my satisfaction					
6.	I can always manage to breastfeed even if my baby is crying					

7.	I can always keep wanting to breastfeed					
8.	I can always comfortably breastfeed with my family members present					
9.	I can always be satisfied with my breastfeeding experience					
10.	I can always deal with the fact that breastfeeding is time consuming					
11.	I can always finish feeding my baby on one breast before switching to the other breast					
12.	I can always continue to breastfeed my baby even when he/she is unwell					
13.	I can always manage to keep up with my baby's breastfeeding demands					
14.	I can always tell my baby is finished breastfeeding					

Observational checklist for assessing breastfeeding technique

S/no.	Variable	Criteria for variables	Yes	No
1.	Positioning	1. Baby's body close to the mothers		
		2. Baby's head and body straight		
		3. Baby's mouth and body are facing the breast		
		4. The whole body supported by mother		
2.	Attachment	1. More areola is seen above the baby's top lip		
		2. Baby's mouth has a wide open		
		3. Lips are flanged out/turned out ward		
		4. Chin is pushed into the breast and the nose is clear of the breast		
3.	Suckling	1. Slow sucks		
		2. Deep sucks		
		3. Sometimes pausing		
