# Supplementary material for: Prevalence and determinants of effective breastfeeding technique among early postpartum mothers in Fuzhou, China: A cross-sectional study
Source: PLoS One. 2025 Feb 25;20(2):e0319408. doi: 10.1371/journal.pone.0319408 (PMC11856331; doi:10.1371/journal.pone.0319408)
Supplement: S2 File — (PDF) [file pone.0319408.s002.pdf]

CHISESE QUESTIONNAIRE

编号：___________

问卷

您好，我是福建医科大学的研究生。我们正在做关于母乳喂养相关课题的研究，十分感谢您抽出时间来填写这份问卷，请根据自身的情况来填写如实填写，
最终结果仅供学术研究，不会泄露您的个人资料，谢谢合作。

恭喜您成为伟大的母亲，祝您身体健康，孩子快乐成长！

问题指导语：本问卷每个问题有几种答案填写要求，请根据问题和答案设置要求，选择或填写与您相符的选项。

第一部分 社会人口学特征

填写说明：请在划线处填上文字，其他设有选项的题目，请直接在合适的选项上√。

1. 您的年龄是____岁（周岁）
2. 您的民族是？

   A.汉族 B.少数民族__ C.其他______
3. 您是否有宗教信仰？

   A.无 B.佛教 C.基督教 D.穆斯林 E.印度教 F.其他______
4. 您目前的婚姻状况？

   A.已婚 B.同居 C.未婚 D.离婚 E.丧偶
5. 您的职业是？

   A.家庭主妇 B.事业单位员工 C.非事业单位/私人公司企业员工

   D.个体经营者 E.学生
6. 您的教育水平是？

   A.没有上过学 B.小学 C.初中 D.高中

   E.中专F.大专G.本科 H.研究生及以上
7. 您的家庭所在地？（居住地/常住地）

   A.农村 B.乡镇 C.城市
8. 您是否是独生子女？

   A.是 B.否
9. 您的家庭人均月收入(元)？(各选项取上限)

A.≤2000　　B.2001-5000 C.5001-10000　　D.10001-30000E.30001-50000
F.≥50001

10.您平常居住的环境？

A.安静　B.一般　　C.嘈杂

11.您现在每天睡眠时间是？(取上限)

A.≤6小时　　B.6-8小时　　C.＞8小时

12.您目前的食欲情况？

A.较差　B.一般　　C.较好

13.您的家庭有几口人？

A.2 人　B.3 人 C.4 人 D.5 人 E.6 人及以上

第二部分 产科和婴儿特点

填写说明：请在划线处填上文字，其他设有选项的题目，请直接在合适的选项上打“√”。

14.包括本次，您一共怀孕________次，生产过________次，流产过________次，现有孩子_个。

本次怀孕，

15.您有没有在医疗保健机构（医院）参加产前保健（产检等）？(若选项为无，则跳过第 3 题)

A.有　　B.无

16.本次怀孕，整个孕期您共参加过几次产前保健？

A.一次　B.两次　　C.三次　　D.四次或更多

17.本次怀孕，整个孕期您有没有参加孕妇学校呢？(若选项为无，则跳过第 5 题)

A.有　　B.无

18.如有参加孕妇学校，关于母乳喂养知识的课程，您参加了几次呢？

A.一次　B.两次　　C.三次　　D.四次或更多

19.您这次怀孕是有计划的吗？

A.是　　B.否

20.您怀孕期间有没有得到相关支持？(包括经济、家人、朋友等)（若选项为否，请跳过第 8 题)

A.是　　B.否

21.您从谁那里得到相关支持？（可多选）

A.配偶　B.爸爸妈妈　C.　公公婆婆　　　D.亲戚，如姐姐、阿姨、哥哥等

E.朋友　　　F.其他________（请补充）

22.您有见过其他人哺乳吗？

A.有　　B.无

23.您怀孕期间有获得过关于母乳喂养技术的知识吗？

A.有　　B.无

24.您是从谁那里得到有关母乳喂养技术的知识？（可多选）（若选项包括A，请填写第 12 题，否则跳过）

A.医疗保健提供者　　B.父母　　　C.丈夫　　　D.亲戚　　　E.朋友

F.其他______（请补充）

25.医疗保健提供者来自哪个行业？（可多选）

A.护士　B.医生　　　C.营养师　　D.其他______（请补充）

26.您本次怀孕的分娩方式是什么？

A.正常分娩　　B.剖宫产　　C.辅助分娩

27.分娩后，您是否立即得到了有关母乳喂养技术的教育？（若选项为否，请跳过第 15 题）

A.是　　B.否

28.您是从谁那里得到的有关教育？

A.护士　B.医生　　　C.营养师　　D.其他______（请补充）

29.您给您的宝宝喂奶之前喂过其他食物吗？如温水、葡萄糖水等(若选项为无，则跳过 17 题）

A.有　　B.无

30.您在母乳喂养前喂食其他食物的原因是？（可多选）

A.乳汁分泌延迟 B.文化习俗　C.医疗机构建议　　D.家人或朋友建议

E.担心宝宝饥饿\口渴　　F.其它________（请补充）

31.您是否给您的孩子补充/辅助喂养？（若选项为否，则跳过 19 题）

A.是　　B.否

32.补充/辅助喂养的理由是什么？（可多选）

A.母乳不足　　B.文化习俗　C.医疗机构建议　　D.担心宝宝营养不足

E.其它____________（请补充）

33.您现在是否存在乳房问题？（若选项为否，则跳过21题）

A.是　　B.否

34.如果是，请说明具体存在的乳房问题是:

A.乳头皲裂　　　　B.乳腺炎　　C.乳房　　　D.乳头凹陷E.其他____（请补充）

35.您宝宝出生时的胎龄是________周

36.宝宝的性别是？

A.男　　B.女

37.出生体重 g

38.现在是宝宝出生后_____小时。

第三部分 母乳喂养技术知识

填表说明：此量表描述您对母乳喂养技术知识的了解情况，请您在方格中您认为合适的答案打“√”。

条目	1 正确	2 不正确	3 不确定
1. 良好的哺乳姿势有益于母婴。			
2. 建议母亲在给婴儿喂奶时，身体前倾，或者将乳房向前推送向婴儿。			
3. 在哺乳期间，可以通过移动手臂将婴儿靠近乳房。			
4. 母乳喂养时，婴儿身体的重要部位需要得到支持，包括头部、颈部和臀部。			
5. 母乳喂养时，母亲可以采取坐、卧、站等姿势。			
6. 婴儿下唇下方可见的乳晕多于上唇上方可见的乳晕，或者上下唇的乳晕数量相等， 这是恰当含接的标志。			
7. 不恰当的含接姿势包括婴儿的下巴远离乳房，或者婴儿的嘴巴没有张开。			

8. 宝宝的上唇和下唇外翻是恰当含接的标志。			
9. 乳头内陷会导致婴儿含接不良。			
10. 哺乳期母亲经历乳头疼痛或乳头皲裂表明婴儿含接姿势不当。			
11. 含接姿势不当的婴儿很可能吮吸无效。			
12. 为了有效地吮吸，婴儿必须慢慢地吮吸，但要深，有时还要停顿。			
13. 婴儿在没有吞咽的情况下一直吮吸的速度很快，这是吮吸有效的标志。			
14. 无效的吮吸是母乳量不足的原因之一。			
15. 乳房出现充血和乳腺炎等问题，大多是由无效的哺乳造成的。			
16. 丰满的乳房通常会导致婴儿无法恰当含接。			
17. 经常哺乳、热敷或按摩可以解决乳房充血的问题。			
18. 不建议在乳房充盈的情况下用母乳喂养婴儿。			

第四部分 母乳喂养自我效能

中文版母乳喂养自我效能简式量表

此量表是描述您进行母乳喂养的自信心状况，请您独立完成，在最接近您感受的数字上“√”，答案没有对错之分。

1=一点儿也没有信心

2=不是很有信心

3=有时有信心

4=有信心

5=非常有信心

		1	2	3	4	5
1	我总能确保宝宝母乳充足					
2	我相信我能够做好母乳喂养，就想以前我总能很好得应付那些自己从来没有做过的事儿一样					
3	我总是能够完全以母乳哺喂而不给孩子添加代乳品					
4	我总是能够确保宝宝在整个吃奶过程中能够正确含住乳头吸吮					
5	我总能将母乳喂养的状况控制到令我满意					
6	即使在孩子哭的时候，我也总能将哺乳进行下去					
7	我总能保持那种想要坚持母乳喂养的愿望					
8	喂奶时即便有家人在场，我也能心情放松而不会感到尴尬					
9	我总是能够满意自己母乳哺育的状况					
10	虽然母乳哺喂比较耗时，我也能够应对					
11	我总能只用一只乳房就把孩子喂饱					
12	每次喂奶我都能一气呵成而不会间断					
13	我总是能够配合孩子对母乳的需求来喂奶					
14	我总是能够判断孩子是否吃饱了					
